# Loss of Sterol Biosynthesis in Economically Important Plant Pests and Pathogens: A Review of a Potential Target for Pest Control

**DOI:** 10.3390/biom14111435

**Published:** 2024-11-11

**Authors:** Paul Dahlin, Andrea Caroline Ruthes

**Affiliations:** 1 Entomology and Nematology, Plant Protection, Agroscope, Müller-Thurgau-Strasse 29, 8820 Wädenswil, Switzerland; 2 Mycology, Plant Protection, Agroscope, Route de Duillier 60, 1260 Nyon, Switzerland

**Keywords:** insects, nematodes, oomycetes, control strategies, biochemical pathway, auxotroph

## Abstract

Sterol biosynthesis is a crucial metabolic pathway in plants and various plant pathogens. Their vital physiological role in multicellular organisms and their effects on growth and reproduction underline their importance as membrane compounds, hormone precursors, and signaling molecules. Insects, nematodes, and oomycetes of the Peronosporales group, which harbor important agricultural pests and pathogens, have lost the ability to synthesize their own sterols. These organisms rely on the acquisition of sterols from their host and are dependent on the sterol composition of the host. It is thought that sterol-synthesizing enzymes were lost during co-evolution with the hosts, which provided the organisms with sufficient amounts of the required sterols. To meet the essential requirements of these organisms, some sterol auxotrophs retained a few remaining sterol-modifying enzymes. Several molecular and biochemical investigations have suggested promising avenues for pest and pathogen control by targeting host sterol composition, sterol uptake, or sterol modification in organisms that have lost the ability to biosynthesize sterol de novo. This review examines the loss of sterol biosynthesis de novo in insects, nematodes, and oomycetes with the aim of investigating the sterol metabolic constraints and sterol acquisition of these organisms. This will shed light on its potential as a control target for the management of sterol-dependent organisms in a comprehensive agronomic approach.

## 1. Introduction

Sterols are amphipathic biomolecules with hydrophilic (polar) and hydrophobic (non-polar) moieties that are synthesized by the monooxygenation of squalene, which is catalyzed by squalene epoxidase and subsequently cyclized by oxidosqualene cyclase to lanosterol or cycloartenol [1,2,3].

The first cyclic product of the sterol biosynthetic pathway, lanosterol in animals and fungi or cycloartenol in plants and algae, shows the diversification (with some variation) between different kingdoms [3,4].

In eukaryotes, this class of isoprenoid-derived lipids performs various essential functions, such as being a component of the cell membrane, stabilizing the phospholipid bilayer, and playing a role as hormone precursors [5,6]. These fundamental biomolecules have also been implicated in the stress response to biotic and abiotic stresses [7,8,9].

**Figure 1:**
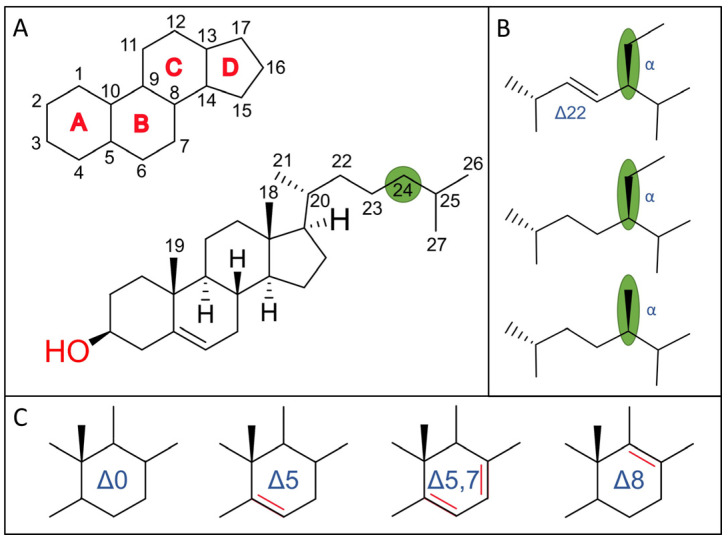
(**A**) Chemical structure of 5α-cholesten-3β-ol (cholesterol). Carbon numbering according to IUPAC-IUB 1989 using the sterane structure, with each ring labeled from A to D. (**B**) The amphipathic side chain at C-17 of phytosterol can be modified at C-24 as highlighted in green, with stigmasterol at the top, β-sitosterol in the middle, and campesterol at the bottom. (**C**) The crucial double bonds in the B-ring are labeled with the delta symbol (Δ).

The end-sterol biosynthetic pathway is thought to be kingdom-specific and to have evolved over time, as the last common eukaryotic ancestor possessed most of the sterol-synthesizing enzymes [2,10]. Mammalian and fungal organisms contain either cholesterol or ergosterol as their primary sterol, whereas plant sterols are characterized by a diverse mixture of C-24 phytosterols [3] (Figure 1). Of the more than 250 different reported sterols and related compounds identified in the kingdom Plantae, β-sitosterol, stigmasterol, and campesterol are the three major sterol components [3,11].

Although there are many forms of sterols and more than 1000 natural sterol derivatives have been reported, they all share a basic sterane framework that forms the core of all sterols [3] (Figure 1A). This core is a tetracyclic C17 hydrocarbon 1,2-cyclopentanoperhydrophenanthrene that can be substituted with methyl groups at the C10 or C13 positions. Attached to this basic tetracyclic ring structure [named rings A, B, C, and D (Figure 1A)], we find a 3β-hydroxyl group at position C3 and a side chain of 8–10 carbon atoms at C-17, giving them their amphipathic nature [3,12,13]. Sterols can also have a methyl or ethyl group at the C24 position with either an α (front) or β (back) stereochemical orientation (Figure 1B). In addition, sterols can be conjugated at the 3β-hydroxyl group with fatty acids, glycosides, or sulfate esters [14]. Finally, kingdom-specific end sterols differ only in minor structural details (Figure 1C) but can be subsequently metabolized and/or substituted.

## 2. Sterol Biosynthesis

**Figure 2:**
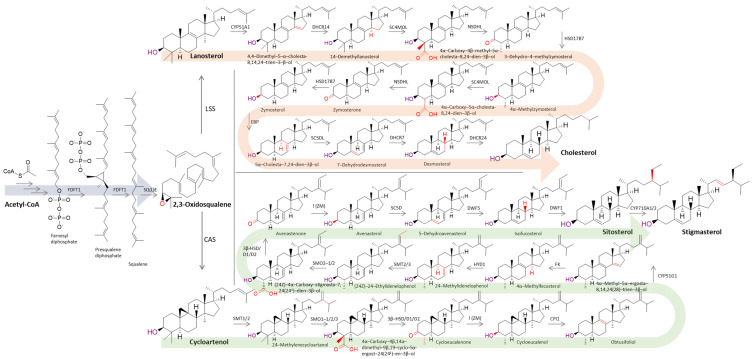
Schematic representation of enzymatic steps from Acetyl-Coa leading to the Δ5 sterol biosynthetic pathway, illustrating the lanosterol (LSS, in invertebrates) biosynthesis route leading to cholesterol and the cycloartenol (CAS, in plants) biosynthesis route leading to stigmasterol. With the exception of FDFT1, farnesyl-diphosphate farnesyltransferase 1, all enzymatic abbreviations and EC numbers for vertebrate and plant enzymes are detailed in Table 1. Each enzymatic reaction is highlighted in red on the corresponding chemical structures. The first cyclic products of each sterol biosynthesis pathway, as well as the final sterols, are highlighted in bold.

Sterol biosynthesis is thought to have evolved after the appearance of oxygen about 2.5 billion years ago [15]. The sterol biosynthesis pathway leading to the different major Δ^5^ sterols, either via the cycloartenol or lanosterol pathway shows close similarities between different kingdoms (Figure 2). The canonical sterol biosynthetic pathway is a multistep process leading to Δ^5^ sterols, comprising 10 (animals) to 13 (plants/fungi) enzymatic steps (outlined in Table 1), starting with the monooxygenation of squalene.

Among all sterol pathway enzymes, oxidosqualene cyclase (lanosterol–cycloartenol synthase) is the most conserved enzyme at the sequence level, and orthologs are present in all organisms capable of de novo sterol biosynthesis via the lanosterol or cycloartenol biosynthetic pathway [10] (Table 1). Remarkably, plants such as *Solanum lycopersicum* have the ability to synthesize cholesterol via the lanosterol or cycloartenol pathway [16]. Although fungi synthesize their end sterol (ergosterol) via the lanosterol synthesis pathway and plants mainly via the cycloartenol pathway, they differ from animals, which use the lanosterol synthesis pathway, by their ability to modify the side chain by C-22 desaturation (1.14.19.41) and C-24/28 methylation (EC. 2.1.1.41/143), which are not present in animals [10]. In fact, there are also sterol auxotrophic multicellular organisms that have lost the ability to synthesize their own sterol and must obtain it for growth and reproduction [10].

## 3. Agricultural Pests and Pathogens with Impaired Sterol Biosynthesis

Since 1935, it has been suggested that insects require a dietary source of sterols or a similar substance for growth and reproduction and are, therefore, sterol auxotrophs [17]. The loss of sterol biosynthesis in certain organisms represents a remarkable evolutionary adaptation. The diverse group of sterol-dependent organisms, oomycetes belonging to the Peronosporales group [18,19,20], nematodes [21,22], and insects (including all other arthropods) [5,17,23], includes a wide range of plant pests and pathogens of agricultural economic importance. For example, the oomycete *Phytophthora infestans* and *Hyaloperonospora arabidopsidis* [24,25], the groups of plant-parasitic nematodes *Meloidogyne* spp. and *Globodera* spp. [26,27] or the insects *Myzus persicae* (peach potato aphid), *Leptinotarsa decemlineata* (Colorado potato beetle), *Thrips tabaci* (onion thrips), and *Plutella xylostella* (diamondback moth) [28] are only a small selection of these economically relevant sterol auxotrophs.

### Loss of Sterol Biosynthesis

Various feeding studies have been carried out to elucidate the missing step(s) in sterol biosynthesis in several families of sterol auxotrophic organisms. For example, *Thermobia domestica*, the firebrat, fed with radioactively labeled acetate, was unable to incorporate these sterol precursors into cholesterol, and it was concluded that the de novo sterol biosynthesis was lacking [29].

Axenic cultures of *Caenorhabditis briggsae*, *Turbatrix aceti*, and *Panagrellus redivivus* supplemented with ^14^C-labeled mevalonate showed that these nematodes were unable to synthesize cholesterol from this sterol precursor [30].

In studies with *C. briggsae*, *Caenorhabditis elegans,* and *T. aceti,* in which acetic acid, DL-mevalonic acid lactone, farnesol, squalene, and lanosterol were added to the culture media as potential sterol precursors, only squalene, lanosterol, and cholesterol showed dose-dependent effects on nematode growth [31]. Therefore, it has been proposed that the metabolic disruption occurs between farnesol and squalene or even before [31]. However, these findings are in contrast to previous studies on the free-living nematode *C. briggsae*, which failed to reproduce when reared on bacteria substituted with squalene, whereas reproduction was supported when supplemented with ergosterol, 7-dehydrocholesterol, β-sitosterol, stigmasterol, and cholesterol [32]. Similar studies on the insects *Dermestes vulpinus* (now *Dermestes maculatus*), *Calliphora erythrocephala*, and *Musca domestica* showed that mevalonic acid and squalene could not replace cholesterol [23].

Feeding studies with oomycetes also showed that the sterol precursor squalene could not subsidize the growth of *P. infestans* compared to supplementation with β-sitosterol, lanosterol, cycloartenol, zymosterol, lathosterol, brassicasterol, desmosterol, stigmasterol, cholesterol, fucosterol, or ergosterol [19].

With the increase in high-quality sequencing data, recent studies have confirmed that sterol auxotrophs in insects, nematodes, and oomycetes lack the enzymes for the synthesis of squalene to lanosterol (Figure 2), namely the enzymes farnesyl-diphosphate farnesyltransferase1 (FDFT1), squalene monooxygenase (SQE), and oxidosqualene cyclase (OSC) [19,20,22,33,34,35]. Although there may be alternative enzymes to squalene oxidase, as predicted for diatoms and brown algae [36], the feeding studies of previous research described above are inconsistent with the hypothesis of the existence of alternative enzymes co-opting these pathways in sterol auxotrophs. Moreover, it is unlikely that other genes would compensate for this function, as individuals may benefit from losing energetically costly enzymatic steps that previously consumed resources for sterol biosynthesis.

## 4. Exploiting the Loss of Sterol Biosynthesis in Plant Pests and Pathogens

The fact that insects, nematodes, and some plant pathogens lack the ability to synthesize sterols de novo makes sterols an important primary nutrient for maintenance, reproduction, and survival. However, the exact nature of sterol auxotrophy remains to be elucidated, and the extent to which dietary sterols and plant sterol composition affect insects, nematodes, and other plant pathogens remains to be determined.

As described above, the requirement of dietary sterol for different physiological processes is essential for all the sterol auxotrophic plant pathogenic organisms that have been studied to date. For example, in the absence of sterols, mycelial growth of the oomycete *P. infestans* is stagnated [19], *Drosophila* larval development is arrested [37], and nematode development is impaired in terms of growth and reproduction [22].

Interestingly, sterol levels appear to have different effects in the nematode *C. elegans* than in *Drosophila*. In *Drosophila*, membrane sterol levels can be dramatically reduced but remain essential [37], whereas *C. elegans* does not accumulate membrane sterols in most tissues [38] and requires sterols primarily for steroid hormone synthesis [38,39].

Understanding how sterols are acquired and utilized by the different plant pests and pathogens is important to better understand whether sterols can be used as potential targets for the control of these important sterol auxotrophic organisms.

### 4.1. Insects

Among sterol auxotrophic organisms, insects are the most studied in an agricultural context. They acquire sterols mainly by parental transfer during oogenesis and from the diet. Sterols are absorbed from the gut lumen through several receptors and transport proteins that regulate their flux into and within cells [40]. Since inadequate uptake and delivery of sterols for oogenesis leads to reduced fertility, sterols play a critical role in the development of offspring.

While plants are predominantly composed of C-24 phytosterols, plant-feeding insects typically have cholesterol as their primary sterol composition [5]. Cholesterol plays several vital roles in insects: it is a structural component of the plasma membrane, acts as a precursor of ecdysone (an important hormone controlling growth and development), and functions as a signaling molecule [5,40,41]. Consequently, many herbivorous insects convert phytosterols to cholesterol because they struggle to obtain sufficient amounts of cholesterol from their diet. For example, insects such as aphids typically convert about 40% of β-sitosterol to cholesterol [42].

**Figure 3:**
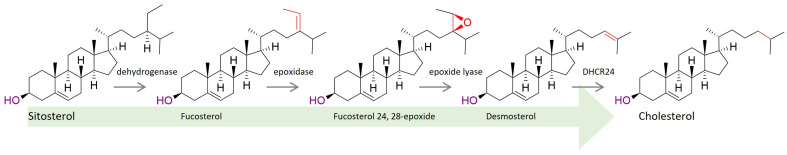
Predicted sitosterol-to-cholesterol conversion pathway, namely the phytosterol dealkylation pathway. Each enzymatic reaction is highlighted in red on the corresponding chemical structures. The enzyme 24-dehydrocholesterol reductase (DHCR24) is the only confirmed reaction within this pathway.

Based on the conversion of phytosterols to cholesterol in various insects, the enzymatic steps have been predicted (Figure 3), but the enzymes responsible have not been studied in great detail. A microsomal membrane-associated reductase from the silkworm *Bombyx mori* mediated the conversion of desmosterol to cholesterol in vitro [43]. Genome-wide studies in *Helicoverpa zea* identified dehydrogenase and epoxidase genes potentially involved in sterol metabolism [44]. Further in silico studies in *Drosophila melanogaster*, *Tribolium castaneum,* and *Tetranychus urticae* confirmed the absence of the first three genes encoding enzymes of the canonical cholesterol synthesis pathway (Supplementary data in Shamsuzzama et al. [22]). The same bioinformatic analysis identified cholesterol synthesis genes homologous to those of the human pathway, namely *CYP51A1*, *LBR*, *SC4MOL, NSDHL*, *HSD17B7*, *SC5D*, and *DHCR7*. These studies were performed in the nematode *C. elegans* and ultimately suggested the existence of a co-opted sterol synthesis pathway for cholesterol, supported by the sterols provided by plants and fungi [22]. However, some herbivorous insects, such as those in the Pentatomomorpha group, have lost the ability to convert phytosterols to cholesterol [45]. These insects appear to have evolved around phytosterols and are able to synthesize the ecdysone makisterone, an important insect molting hormone, from C28 sterols that differ from the other C27 molting hormones by a C-24 methyl group [45,46].

Even if herbivorous insects are exposed to minimal levels of cholesterol, have circumvented the need to produce ecdysone from cholesterol, or have developed the ability to replace membrane cholesterol with other sterols, the lack of these capabilities can prove fatal for some of them [47]. In particular, Δ^7^ (D7) sterols have been reported to delay insect development and increase mortality in non-specialist herbivorous insects, although certain insects, such as *Drosophila pachea,* can metabolize them [47,48,49].

Early biochemical studies also suggested that certain grasshoppers lack the ability to convert Δ^7^ sterols to Δ^5^ sterols. In addition, the presence of a double bond at position C22 inhibits the removal of the alkyl group at C-24, thereby affecting the physiological responses of the grasshoppers when they consume Δ^7^ sterols or Δ^22^ sterols [50]. A review by Behmer [51] confirmed that mixed sterols diets can have negative effects on herbivorous insects, especially when the sterols have an alkyl group at the C-24 position.

Since insects cannot convert all types of “plant” sterols, there is potential for targeted modification of plant sterols as a method of insect control. For example, genetically modified tobacco plants carrying the 3-hydroxysteroid oxidase (*choM*) gene from *Actinomyces* sp. A19249 produce an atypical sterol composition, mainly featuring ketone steroids such as the cholesterol derivative, cholest-4-en-3-one. These plants have been shown to reduce reproduction and increase mortality of the aphid *M. persicae* [52]. Similarly, a study using transgenic tobacco plants enriched in atypical plant steroids, mainly stanols and ketone steroids, affected the reproduction of economically important caterpillars such as *Heliothis virescens*, *Spodoptera exigua*, and *Manduca sexta* after the second generation fed on the plants [53].

Furthermore, RNAi-mediated knockdown of the Δ^8^-Δ^7^ sterol isomerase gene (*HYD1*) significantly impaired the growth and survival of diamondback moth (*P. xylostella*) larvae reared on *A. thaliana* plants modified to contain sterols with a Δ^8^ configuration [54]. The reduced growth and survival were attributed to a >95% reduction in HYD1 transcripts, resulting in an accumulation of Δ^8^ sterols and a lack of dietary cholesterol.

### 4.2. Nematodes

Plant-parasitic nematodes are obligate plant parasites that cannot complete their life cycle without their host. Until now, no plant-parasitic nematode has been able to feed on artificial/controlled media, and therefore, sterol studies on nematodes have mainly been carried out on the model nematode *C. elegans* or its relatives. However, knowledge of the sterol composition of plant-parasitic nematodes has been used to predict the metabolic capacity of plant-parasitic nematodes by comparing nematode sterols with host sterol composition [55]. For example, studies of *Ditylenchus dipsaci* feeding on alfalfa callus tissue showed that the major nematode sterols were cholesterol and lathosterol, as opposed to plant sterols such as 24-ethylcholesta-7,22-dienol and 24-ethyllathosterol [56]. Similar dealkylation of plant C24 sterols to cholesterol has been reported for *Rotylenchulus reniformis* feeding on *Gossypium hirsutum* [57] and for *Heterodera zeae* propagated on *Zea mays* [56]. In addition, primary metabolic sterol modifications in certain plant-parasitic nematodes involve the saturation of the phytosterol core, as has been noted in the root-knot nematodes *Meloidogyne arenaria* and *Meloidogyne incognita* and the root lesion nematode *Pratylenchus agilis* [56].

A study examining the sterol composition of the tobacco cyst nematode *Globodera tabacum* while feeding on “N.C. 95” tobacco plants revealed a composition consisting of 43% 24-methylcholestanol, 20% 24-ethylcholest-22(E)-enol, 12% cholesterol, 9% 24-methylcholesterol, and 8% 24-ethylcholesterol. Although direct analysis of host plant roots was not performed, it was inferred from the sterol profiles of higher plants, which typically have minimal stanol content, that *G. tabacum* may have the ability to hydrogenate Δ^5^ bonds [58]. Subsequently, it was found that C-4 sterol methylation catalyzed by the enzyme STRM-1 in *C. elegans,* which is involved in the dafachronic acid (DAS) synthesis, was found to be nematode-specific [59].

Over the years, phytosterol modification, especially of β-sitosterol to cholesterol, has been confirmed and described in *C. elegans* [22,60]. Shamsuzzama et al. [22] even reported that *C. elegans* is able to grow on all intermediates of the sterol synthesis pathway, starting with lanosterol. However, no growth was observed in nematodes grown on the sterol precursor [22].

Interestingly, although *C. elegans* is not a plant-parasitic nematode, it relies on the dealkylation of β-sitosterol as a critical process for the synthesis of cholesterol. The enzyme 24-dehydrocholesterol reductase (DHCR24) is involved in this process. Deficiency of this enzyme results in impaired growth of the *C. elegans tm2141* mutant, which lacks phytosterol dealkylation activity [60]. In addition, “atypical” sterols also affect the physiological function of the nematode. For example, when *C. elegans* were fed clerosterol, a sterol found in higher plants that lack the necessary C24 or C25 hydrogens for dealkylation by DHCR24, the nematodes were unable to convert it into cholesterol, leading to developmental failure [60]. Similar results were obtained when azasteroids, in which a nitrogen atom replaces a carbon atom in the steroid ring structure, inhibited ∆^24^-sterol reductase and impaired growth and reproduction in *C. elegans* [55]. Thus, the 24-dealkylation pathway (Figure 3) appears to be essential for the processing of phytosterols in nematodes. However, the interpretation of sterol studies using *C. elegans* as a model can be misleading. This is because the curated WormBase database indicates that plant-parasitic nematodes such as *Meloidogyne*, *Globodera*, and *Ditylenchus* lack some of the sterol-related enzymes predicted in *C. elegans*, as described by Shamsuzzama et al. [22]. In addition, according to the BUSCO annotation and assembly score on the WormBase ParaSite home page [version: WBPS18 (WS285)-archive: WBPS17], which identifies single-copy orthologs present in over 90% of animals, *C. elegans* scores over 99%. In contrast, the obligate plant-parasitic nematode *M. incognita* achieves a BUSCO score of 75.4%. Given these relatively recent sequencing results, we hypothesize that obligate phytoparasitic nematodes may have lost more genes compared to the free-living nematode *C. elegans*.

Interestingly, nematodes may possess another type of sterol carrier enzyme that could facilitate the uptake of sterols from their hosts. Recently, a unique protein, venom allergen-like protein with a conserved CAP (cysteine-rich secretory proteins/antigen 5/pathogenesis-related 1) domain capable of sterol uptake was discovered. However, further investigation is needed to fully understand its role [61,62].

### 4.3. Oomycetes

Sterol auxotrophy in oomycetes is often highlighted to distinguish them from fungi, especially with respect to the loss of sensitivity to azole fungicides that target the CYP51 enzyme in the sterol biosynthetic pathway and is used as a crop protection measure. Functional and molecular studies emphasized that only oomycetes belonging to the group Peronosporales are sterol auxotrophs [19]. Interestingly, within the group of oomycetes, the Saprolegniales can synthesize sterols via the lanosterol biosynthetic pathway [18,19] to fucosterol (*Aphanomyces euteiches*), desmosterol, 24-methylenecholesterol, and/or cholesterol (*Saprolegnia parasitica*) [63,64]. Conversely, oomycetes of the Peronosporales group are dependent on host sterols for their growth and reproduction. Some oomycetes within the Peronosporales clade have retained parts of the ancestral sterol biosynthetic pathway in their genomes. For example, *Phytophthora* and *Pythium* still possess homologs of ERG3 and DHCR7 encoding C-5 sterol desaturase (EC 1.14.19.20) and Δ^7^-sterol reductase (EC 1.3.1.21), respectively [19,20]. However, *H. arabidopsidis*, the causal agent of downy mildew in *Arabidopsis thaliana*, appears to have lost these genes, possibly related to its obligate phytopathogenic life cycle [20].

**Figure 4:**
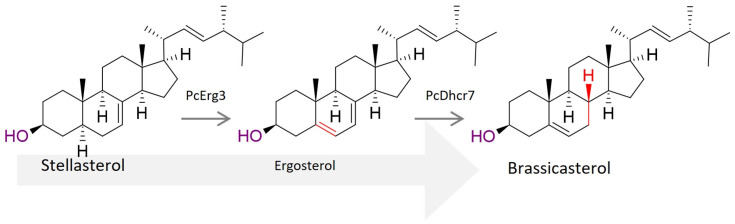
Predicted (stellasterol)ergosterol-to-brassicasterol conversion pathway through the enzymes PcErg3 (C-5 sterol desaturase) and PcDhcr7 (Δ7-sterol reductase). Each enzymatic reaction is highlighted in red on the corresponding chemical structures.

In the case of *Phytophthora capsici*, the Δ^7^-sterol reductase PcDhcr7 showed sterol reductase activity when heterologously expressed in *Saccharomyces cerevisiae* and converted ergosterol to brassicasterol (Figure 4) [65]. This finding is important because sterol studies with *Phytophthora cactorum* have shown a possible conversion of Δ^5,7^ sterols to Δ^5^ sterols, presumably by DHCR7 [66]. Further analysis of *PcDhcr7* revealed its role in zoospore pathogenicity and mycelial development, demonstrating that this gene has multiple essential functions.

ERG3, the C-5 sterol desaturase in *P. capsici* (PcErg3), retained its activity and was shown to convert stellasterol to the downstream product brassicasterol via ergosterol and the enzymatic function of the Δ^7^-sterol reductase PcDHCR7 (Figure 4) [67]. However, by deleting the PcErg3 gene, the study concluded that this enzyme is not obligate for *P. capsici* and has no essential function in host–pathogen interaction compared to PcDhcr7 [65,67]. Moreover, it has been suggested that sterol composition and sterol preferences have co-evolved between host and pathogen, given the variation in sterol composition among different plant species [20]. Nevertheless, the mechanism of sterol recruitment in oomycetes remains poorly understood. It is proposed that elicitin (ELI) and elicitin-like (ELL) proteins act as sterol carriers in oomycetes. These small, secreted proteins share a highly conserved 98 amino acid domain that forms a hydrophobic cavity capable of accommodating sterols and fatty acids [68,69]. Whether the ELL protein actually functions as a sterol carrier remains unclear. However, it is noteworthy that ELIs are exclusively found in the sterol auxotrophic group of Peronosporales [70]. While it has not been investigated whether these ELI, ELL, or other sterol carrier proteins selectively recruit specific phytosterols, *P. cactorum* showed a preference for Δ^5^ sterols over Δ^5,7^ sterols [66], suggesting a possible selective mechanism behind sterol uptake.

Certain phytosterols also had a negative effect on oomycetes of the Peronosporales group. For example, the reproductive success of *Plasmopara viticola* on the grapevine *Vitis vinifera* was altered after host plant sterols were modulated with azole fungicides (demethylation inhibitors) [71]. When *Phytophthora sojae* was grown on sterols from its host plant *Glycine max* (soybean), which contained a mixture of 13 major sterols that differed in composition between seeds and shoots, it was shown that seed sterols containing a C4 methyl group, such as cycloartenol, were not taken up. Oospore production was impaired, and oomycete growth was significantly affected, especially at a β-sitosterol/cycloartenol ratio of 30/70 [72].

## 5. Pathogen Life Style and Gene Loss

Sterols such as ergosterol, which are found in fungal cell membranes, can trigger characteristic elicitation steps in plants. Plants recognize ergosterol as a pathogen-associated molecular pattern and initiate a cascade of plant defense responses [73,74]. It is hypothesized that to overcome this plant–pathogen molecular dialogue, some organisms, such as *Phytophthora* and *Pythium*, have uncoupled sterol biosynthesis [75] and benefit from obtaining these essential molecules in a different way. In contrast, the black queen hypothesis explains gene loss when essential resources are sufficiently available from other organisms (leaky “helpers”) to cause the loss of a biological function or even of the gene(s) [76]. Therefore, these organisms will depend on these leaky “helpers” (in our case, the plants) to provide the essential products to the community. Since insects have more mobility and feeding options compared to oomycetes or plant-parasitic nematodes, we hypothesize that obligate plant pathogens may have adapted even more to their host and its sterols. This may involve the loss of more sterol-related enzymes. For example, the obligate plant pathogenic oomycete *H. arabidopsidis* appears to have lost all genes involved in sterol biosynthesis compared to the saprophytic species *P. infestans* or *Phytophthora ramorum* [20]. In addition, we hypothesize that plant-parasitic nematodes may have lost more sterol-related enzymes during co-evolution with plants compared to free-living nematodes such as *C. elegans,* which primarily feed on bacteria, yeast, and other microorganisms. This assumption is consistent with the finding that plant-parasitic nematodes share only four homologous genes involved in sterol biosynthesis, as identified by BLAST searches. These genes include a putative 3β-hydroxysteroid-4α-carboxylate 3-dehydrogenase (EC 1.1.1.170), a sterol C8 isomerase (EC 5.3.3.5), a sterol Δ^7^ reductase (EC 1.3.1.21), and a sterol Δ^24^ reductase (1.3.1.71/72) [77]. In contrast, Shamsuzzama et al. [22] identified several human homologous genes responsible for the conversion of farnesyl pyrophosphate to cholesterol by studying the free-living nematodes *C. elegans* (seven homologous genes) and *C. briggsae* (eight homologous genes), as well as insects such as *D. melanogaster*, *T. castaneum*, and *T. urticae*, as detailed in the insect section of this review. However, further molecular and biochemical analyses are needed to confirm this hypothesis.

## 6. Role of Plant-Pathogen Interactions in Sterol Acquisition

The distribution of the more than 250 reported plant sterols varies significantly among plant families, species, and plant organs [3,9,14]. Although β-sitosterol, campesterol, and stigmasterol are the most common plant sterols, their availability can be influenced by an organism’s feeding habits. For example, in tobacco, beans, and Chinese cabbage, cholesterol is the most abundant sterol in the phloem sap, making it an important sterol for phloem-feeding insects [52,78]. Conversely, the pollen of plants has a diverse sterol composition across species. This suggests that the sterol composition of pollen has likely evolved separately in different plant species over time and is not necessarily adapted to the nutritional needs of bees or other pollinating insects, as previously suggested [79]. On the other hand, oomycetes proliferate in different host plant tissues, suggesting the potential for sterol redistribution according to their needs.

Sedentary nematodes acquire sterols from their established feeding sites and are, therefore, dependent on the local sterol source. Studies on *M. incognita* have shown that in *Cucumis sativus* and *S. lycopersicum,* cholesterol was significantly increased in the vicinity of the feeding site (galls). This could indicate an accumulation of cholesterol due to its selective uptake by the nematodes or the conversion of phytosterol to cholesterol by *M. incognita*. However, the most significant changes in plant sterol during *M. incognita* infection were the increase in β-sitosterol and the decrease in stigmasterol, possibly facilitated by the C22-desaturase gene *CYP710A11* [9,80]. This would be consistent with the previously described studies in *C. elegans* that β-sitosterol can be converted to cholesterol. However, a detailed investigation of how sterol-dependent organisms alter the composition of plant sterols to suit their needs remains largely unexplored. To the best of our knowledge, there are no studies demonstrating that these organisms deliberately alter the sterol composition of the host for their own requirements. It is speculated that changes such as the conversion of β-sitosterol to stigmasterol may be a response to biotic stress [81].

## 7. Practical Implications of a Potential Target for Pest Control

In the agrochemical industry, sterol biosynthesis inhibitors are the most important specific chemicals used worldwide to control fungal plant diseases [82]. While the azole subclass, including imidazole and triazole, has been extensively studied for their role as inhibitors of sterol 14α-demethylase in the control of fungal diseases, less attention has been paid to exploring sterol-related approaches in organisms that rely on external sources of sterols rather than synthesizing their own. Exploring this aspect may offer a promising strategy for pest and pathogen control. To date, there is evidence suggesting that altering the composition of plant sterols can hinder the growth and reproduction of these organisms by depriving them of necessary sterol substrates.

As an increasing number of complete genomes become available, more detailed research can be conducted to understand the loss of sterol-synthesizing enzymes and the pathways that organisms use to convert/coopt sterol biosynthesis. However, detailed studies, both in vitro and in vivo, are still needed to confirm the functions of these enzymes. The study of the enzymes involved in sterol biosynthesis has the potential to pave the way for a targeted approach. In particular, since phytosterol modification appears to be an essential step for insects [51], nematodes [60], and oomycetes [20,65,67], inhibition of the target enzymes could be used as a potential method to control these pests and pathogens. In addition, there is limited research on sterol uptake, in particular, whether certain organisms have the ability to selectively take up cholesterol from a large pool of phytosterols. This is important because cholesterol appears to be critical for hormone synthesis in some organisms.

### 7.1. Chemical Compounds as Suppressors of Sterol Biosynthesis

Inhibition of sterol biosynthesis is an area that is still greatly understudied. However, a number of compounds are known to inhibit sterol biosynthesis. In the agriculture field, triazoles, imidazoles, morpholines, piperidines, pyrimidines, spironolactone, and amorolfine are some of the well-known inhibitors of sterol biosynthesis [83]. These compounds are highly valuable in agriculture, especially for the control of fungal diseases in a wide range of crops. However, the efficacy of sterol inhibitors against insects, plant-parasitic nematodes, and oomycetes is generally limited.

Typically, sterol inhibitors are not used to control insect pests due to their sterol auxotrophic nature. Insecticides usually target the nervous system, growth regulators, or other vital biological processes in insects. The same is true for plant-parasitic nematodes, and most nematicides used to manage nematode populations also act by disrupting their nervous system or other vital functions.

Unlike fungi, oomycetes do not synthesize ergosterol, the target of most sterol inhibitors, making typical sterol biosynthesis inhibitors ineffective. Some specific fungicides appear to be effective against oomycetes of the Peronosporales group, such as phenylamides and carboxylic acid amides. However, these compounds are effective against these oomycetes by targeting different pathways other than sterol biosynthesis. Phenylamides inhibit rRNA biosynthesis [84], while cellulose synthase is postulated to be the primary target enzyme for carboxylic acid amides activity [85].

Essential oils appear to have similar effects to the positive lanosterol synthase (LSS) inhibitor Ro 48-8071. These oils effectively reduce intracellular lipid levels and cholesterol synthesis [86]. However, it is noteworthy that lanosterol synthase is absent in sterol auxotrophic organisms such as insects, nematodes, and oomycetes of the Peronosporales group, which are the target organisms of our review.

Several enzymes have been reported to be affected by γ-lactam alkaloid derivatives. However, no specific enzyme has been characterized to date [87]. The closest enzyme potentially targeted by γ-lactam, is the putative 3-oxo-5-α-steroid-4-dehydrogenase (EC 1.3.99.6), which is not known to play a role in the Δ5 sterol biosynthetic pathway.

Looking closely at the key enzymatic steps of the sterol biosynthetic pathway (Figure 2), compounds that alter 7-dehydrocholesterol reductase (DHCR7) (aripiprazole, cariprazine, haloperidol, trazodone, fentanyl, etc.), 24-dehydrocholesterol reductase (DHCR24) (amiodarone, 24(s),25-epoxycholesterol, TASIN, SH42, U1866A), sterol-C4-methyl-oxidase-like (SC4MOL) (eugenol, CW4142, diazoborines) and cytochrome P450 family 51 subfamily A member 1 (CYP51A1) (fluconazole, ketoconazole) activity [86] would be of particular interest since genes homologous to those of the human pathway have been identified in some insects, nematodes, or oomycetes using bioinformatics tools. However, these compounds have been studied mainly in the medical field and not in the agricultural field. Since our target organisms are plant pests and pathogens, and plants also have homologs of the same genes, extensive studies would have to be conducted to exclude the negative effects of such compounds in the plant sterol biosynthesis pathway, especially in the case of obligate plant pests such as plant-parasitic nematodes.

### 7.2. Biocontrol Agents as Suppressors of the Sterol Biosynthesis

The sterol biosynthesis pathway is not a common target for biocontrol agents used against insects, plant-parasitic nematodes, and oomycetes of the Peronosporales group. However, some biocontrol strategies may indirectly affect the sterol metabolism of such organisms.

Entomopathogenic fungi, such as *Metharhizium robertsii* and *Bauveria bassiana*, are biocontrol agents that infect and kill insects. Although they do not directly inhibit sterol biosynthesis, the infection process can disrupt several metabolic pathways, including those related to lipid metabolism in insects [88,89]. Similarly, entomopathogenic nematodes, such as *Steinernema* spp. and *Heterorhabditis* spp., release symbiotic bacteria (e.g., *Photorhabdus* sp. or *Xenorhabdus* sp.) that disrupt insect metabolism and may indirectly affect sterol processing [90,91].

In the case of nematodes, nematophagous fungi, such as *Purpureocillium lilacinum* and *Pochonia chlamydosporia,* are just some of the fungi known to parasitize the eggs and juveniles [92,93]. Although they do not directly inhibit sterol biosynthesis, their mode of action can disrupt nematode development and metabolism, potentially affecting sterol utilization. Bacteria, such as *Bacillus firmus* and *Pasteuria penetrans*, are other well-known biocontrol agents used to manage plant-parasitic nematodes [94,95]. The bacteria parasitize nematodes, weakening them and possibly indirectly disrupting their sterol metabolism by affecting overall nematode health and development.

Biocontrol agents that target oomycetes include some bacteria, such as *Pseudomonas fluorescens* [96] and *Bacilus subtilis* [97], and fungi, such as *Trichoderma harzianum* [98,99], to name a few. As with insects and nematodes, the biocontrol agents do not directly affect sterol biosynthesis, but they can disrupt related pathways or interfere with membrane integrity. *P. fluorescens* and *B. subtilis* produce secondary metabolites (phenazines and lipopeptides) that can disrupt the cell membrane, not directly by targeting sterol biosynthesis but by compromising membrane integrity, which affects the overall sterol balance. While *T. harzianum* produces enzymes and secondary metabolites capable of degrading cell walls and disrupting cellular processes, indirectly affecting sterol-related functions.

Therefore, while inhibition of sterol biosynthesis by biocontrol agents has greater potential in organisms capable of de novo sterol biosynthesis, such as plant pathogenic fungi, direct inhibition of the sterol biosynthesis pathway is uncommon in biocontrol agents for auxotrophic organisms. As discussed above, some biocontrol organisms may indirectly disrupt the sterol metabolism or related pathways by targeting other vital pathways or cellular structures. In this way, most biocontrol agents achieve a broader disruption of cellular processes, including effects on sterol metabolism.

### 7.3. Feeding with Altered or Rationally Designed “Plant” Sterols

Since mammals, insects, and nematodes take up sterols in different proportions, it is expected that their requirements can be directed to different functions. However, hormone management appears to be a central function in all these organisms, and it has been suggested that altering phytosterol composition may reduce or control sterol-dependent organisms. Studies in insects, nematodes, and oomycetes have shown that atypical sterols can negatively affect their growth and reproduction. Therefore, exploring methods to alter the sterol composition of host plants could be an effective control strategy that should be further investigated.

Various techniques such as transformation (cloning), RNA interference (RNAi), and the CRISPR/Cas9 system have been used to modify plant sterol composition by transformation and/or genome editing [52,54,100,101,102]. These approaches have shown promising results. For example, RNAi-mediated modification of plant sterols in *Arabidopsis* plants has shown efficacy in controlling insects such as the diamondback moth *P. xylostella* [54]. Similarly, genetically modified tobacco plants with altered sterol composition (containing mainly ketone steroids) reduced reproduction and caused high mortality in the aphid *M. persicae* [52].

Modification of sterol composition using plant sterol inhibitors has been found to negatively affect the reproductive success of the oomycete *P. viticola* [71]. However, it is important to consider that modification of plant sterol composition can negatively affect plant growth and development [103,104], as well as potentially affect beneficial insects such as pollinators. Therefore, a thorough investigation of sterol modification is necessary to prevent crop failure, taking into account both biotic and abiotic factors that affect plant performance.

In addition, there is a risk of “misapplication” when using sterol synthesis inhibitors, where, e.g., leaf coverage may not result in the intended sterol modification in all plant organs or may affect non-target organisms in the soil. Consideration should also be given to whether or not main or cover crops that are not intended for human consumption should be studied and used as negative sterol host plants.

### 7.4. Blocking Sterol Uptake

The family of sterol carrier proteins are intracellular proteins responsible for the transfer of sterols and other lipids. Different types and homologs of these proteins have been isolated from different groups of organisms, including insects, oomycetes, and nematodes [61,70,105,106].

Blockade of sterol binding and transport in insects has been shown to reduce the available pool of sterols, potentially affecting cholesterol metabolism [107]. Sterol carrier protein (SCP) 2 has been implicated in lipid binding and transfer functions and can be inhibited by an SCP-2 competitive inhibition mechanism, such as the use of α-mangostin, α-amyrin, quercetin, curcumin, SCP inhibitor (SCPI)-1 (C_17_H_13_Cl_2_N_3_OS), and SCPI-2 (C_19_H_17_ClN_2_OS_2_) [107]. However, the specificity of these compounds needs to be studied in detail to avoid unintended effects on beneficial organisms. In addition, it is important to understand whether these sterol carrier protein inhibitors reach the target organisms in plants, particularly those with an endoparasitic lifestyle.

Nematodes possess a group of genes that encode venom allergen-like proteins with a conserved CAP (cysteine-rich secretory proteins/antigen 5/pathogenesis-related 1) domain, which allows them to capture small hydrophobic molecules such as cholesterol during infection [61,62]. The role of these proteins in sterol sequestration by plant-parasitic nematodes still requires further investigation, particularly whether they can be actively targeted within the nematode feeding site in the host plant.

As described, in oomycetes, a conserved group of sterol carrier proteins called elicitins facilitates sterol acquisition from the host plant [70]. However, elicitin-mediated sterol acquisition has been reported to be inhibited by tannins, a group of polyphenolic compounds, which successfully reduced the functionality of elicitins against *P. ramorum* [108].

### 7.5. RNA Interference Targeting Genes Involved in Sterol Metabolism and Uptake

The use of RNAi to control pests or pathogens can be achieved by a variety of methods. These include host-induced gene silencing (HIGS) using transgenic plants, spray-induced gene silencing (SIGS) using exogenous application of dsRNA (double-stranded RNA), or virus-induced gene silencing (VIGS) using viruses as novel vectors to produce dsRNA inside the organism.

Although these methods have not directly targeted sterol-related genes of pathogens, their potential has been described for oomycetes such as *H. arabidopsidis*, *P. capsici*, *P. infestans,* and *P. sojae* [109,110], insects such as striped stem borer *Chilo suppressalis*, and the pea aphid *Acyrthosiphon pisum* [111,112], or the nematode *M. incognita* [113,114,115] targeting other essential genes.

The closest study using RNAi on a sterol-related gene in *M. incognita* was performed on Mi-sbp-1, a homolog of sterol-binding protein-1 in *C. elegans*, which is a transcriptional regulator of several lipogenesis enzymes, making it a potential target for the management of plant-parasitic nematodes [116]. In this context, RNAi shows significant potential in targeting enzymes involved in sterol modification or uptake.

## 8. Perspective and Future Directions

Sterol biosynthesis stands out as an important target for organisms that depend on these vital molecules, yet it is highly understudied in this regard. As sterols are also essential for organisms lacking de novo sterol biosynthesis, there is great potential for the regulation of sterol-dependent plant pests and pathogens such as insects, nematodes, and oomycetes within the Peronosporales group. Basic research has already demonstrated functional applications with promising potential to support these specific control methods.

From a scientific perspective, it will be essential to better understand the complex relationship between sterols and the biology of sterol auxotrophic organisms to provide valuable insights for the development of more targeted and applicable control strategies as outlined above. Therefore, further research is needed to uncover additional mechanisms that govern the biological action of sterols and to refine existing methods or develop new ones to target these important molecules or the enzymes that modify these molecules by organisms for their specific purposes.

This pursuit may involve altering the sterol composition of host plants, targeting metabolic enzymes of the organisms, or modulating essential sterol uptake mechanisms. These efforts may reveal multiple targets for controlling one or more sterol auxotrophic organisms within a unified strategy. However, future research and agricultural practice will need to explore whether altering host sterol composition can effectively target multiple sterol-dependent organisms in different kingdoms.

In the early 1970s, Heftmann [117] proposed that plant sterols may function similarly to animal sterols and that altering plant sterol composition could affect crop yield. Consequently, modifying host plant sterols, especially for cover crops, may be feasible, potentially reducing populations of plant-parasitic nematodes. However, this approach may not always control airborne pests, such as the Colorado potato beetle, that feed on plants with natural sterol composition.

In this context, RNAi interference targeting genes involved in sterol metabolism and uptake appears to be a potentially more promising approach. In contrast to altering host plant sterols, RNA interference delivery may have fewer compromises, and its specificity can be precisely tailored to the target organisms.

However, regulatory challenges associated with the use of transgenic plants with altered sterol profiles or the delivery of RNAi targeting sterol-modifying enzymes may hinder near-term adoption. Nevertheless, it is worth noting that changes in sterol composition could potentially have the same effect on sterol-dependent organisms. Thus, the search for plants with an altered sterol composition or the modification of plant sterol profiles could serve as an integrated approach to control not only one group of these organisms but also potentially affect the performance of other sterol-dependent organisms.

In addition, as the number of complete genomes sequenced and studied in detail continues to grow, researchers are still uncovering the molecular mechanisms underlying the loss of sterol biosynthesis in insects, nematodes, and oomycetes. This progress will further elucidate the conversion and co-optation pathways involved in sterol biosynthesis and contribute to a deeper understanding of the biology of these organisms.

## Figures and Tables

**Table 1:** Comparison of homologous enzymes of the Δ^5^ sterol biosynthetic pathway from Vertebrates (*Homo sapiens*), Fungi (*Saccharomyces cerevisiae*), and Plants (*Arabidopsis thaliana*). ! Indicates that the enzyme carrying out the C-3 ketoreduction in plants is assumed to be a cycloeucalenone reductase (ZM), as the reaction is predicted to be involved in sterol metabolism by biochemical approaches. NA, not available, indicates that no homolog has been found in the genome of the organism (vertebrates or fungi). Enzyme Commission (EC) numbers are given for each reaction.

	V	F	P	Steps/Function	Enzyme Name	EC-Number
0	SQLE	ERG1	SQE2	Squalene monooxygenation	Squalene monooxygenase	1.14.99.7
1	LSS (7B)	ERG7 (7B)	CAS (7B)	Oxidosqualene cyclization	Lanosterol/Cycloartenol synthase	5.4.99.7/8
2	CYP51A1	ERG11	CYP51G1	C32-Elimination/C14 Demethylation	Sterol 14α-methyl demethylase (CYP51)	1.14.14.154/1.14.13.70
3	DHCR14	ERG24	FK (TM7SF2)	C14-Reduction	Sterol Δ^14^-reductase	1.3.1.70
4	SC4MOL	ERG25	SMO1-1/2/3 SMO2-1/2	C4-Methyl oxidation	Sterol C4-methyloxidase	1.14.13.72/1.14.18.9
5	NSDHL	ERG26	AT3βHSD/D1/2	C3 Dehydrogenation/C4 Decarboxylation	3β- hydroxysteroid- 4α- carboxylate 3- dehydrogenase (decarboxylating) (3β-HSD)	1.1.1.170
6	HSD17B7	ERG27	! (ZM)	C3-Ketoreduction	3β-Keto-reductase (Cycloeucalenone reductase)	1.1.1.270
7	EBP	ERG2X	HYD1	Δ^8^-Δ^7^ Isomerization	Sterol C8 isomerase (EBP)	5.3.3.5
8	XDWF7	ERG3	SC5D	Δ^5^-Desaturation	Sterol C5-desaturase	1.14.19.20/1.14.21.6
9	DHCR7	NA	DWF5	Δ^7^-Reduction	Sterol Δ^7^ reductase (DHCR7)	1.3.1.21
10	DHCR24	ERG4	DWF1	Δ^24^-Reduction	Sterol Δ^24^ reductase	1.3.1.71/72
11	NA	NA	CPI1	9β, 19β-cyclopropane ring opening	Cycloeucalenol cycloisomerase	5.5.1.9
12	NA	ERG5	CYP710A	C22-Desaturation	Sterol C22 desaturation	1.14.19.41
13	NA	ERG6	SMT1/2/3	C24/28-Methylation	Sterol C24-methyltransferase/24-sterol methyltransferase	2.1.1.41/143

## Data Availability

No new data were created, and all information is available within the cited references.
